# Abernethy Malformation: Possible Diagnosis for Patients with Congenital Heart Disease and Persistent Cyanosis

**DOI:** 10.21470/1678-9741-2022-0110

**Published:** 2023

**Authors:** Marilia Maroneze Brun, Mariana Rodero Cardoso, Bruna Cury Borim, Carlos Henrique De Marchi, Ulisses Alexandre Croti

**Affiliations:** 1 Pediatric Cardiology and Cardiovascular Surgery, CardioPedBrasil – Hospital da Criança e Maternidade de São José do Rio Preto, São José do Rio Preto, São Paulo, Brazil.

**Keywords:** Cardiomegaly, Cyanosis, Congenital Heart Defects, Hepatopulmonary Syndrome, Computed Tomography Angiography

## Abstract

**Clinical data:**

Infant, nine months of age, female, diagnosed with congenital heart disease, with signs of heart failure associated with cyanosis and difficulty in gaining weight.

**Chest radiography:**

Cardiomegaly with prevalence of pulmonary vascular network.

**Electrocardiogram:**

Ectopic atrial rhythm with right ventricular overload and left anterosuperior divisional block.

**Echocardiogram:**

Single atrium with absent interatrial septum, atrioventricular connection with a single valve and two orifices, with increased pulmonary pressure and high Qp/Qs.

**Computed tomography:**

Absence of portal vein and intrahepatic segment of the inferior vena cava. Infrahepatic portion continuing with the azygos system at the level of the thoracic cavity, presence of mesenteric-caval communication associated with signs suggestive of hepatic peribiliary fibrosis.

**Diagnosis:**

Abernethy malformation is a rare condition and represents an extrahepatic portosystemic shunt that develops between the mesenteric-portal vasculature and the systemic veins. It may be associated with cardiac malformations and advance with pulmonary hypertension and even the need for liver transplantation. Persistent cyanosis after corrective surgery led to a deeper investigation and correct diagnosis of this malformation.

**Operation:**

Sternotomy with 68 minutes of cardiopulmonary bypass and nine minutes of total circulatory arrest. In the postoperative period, persistence of cyanosis was evident, even though there were no immediate complications. Patient was discharged on the 10^th^ postoperative day. An abdominal computed tomography angiography confirmed the diagnosis of Abernethy type I malformation, and the patient was transferred for liver transplantation after congenital heart disease treatment.

## CASE PRESENTATION

### Clinical Data

A nine-month-old female infant was transferred to our service due to heart murmur and difficulty in gaining weight. She was diagnosed in her native city with congenital heart disease and started on furosemide and digoxin.

Good general condition, peripheral oxygen saturation (O2) of 81%, systolic regurgitant murmur 3+/6+ at middle left sternal border, dyspnea with respiratory rate of 50. Medialized liver on palpation. Present and symmetrical peripheral pulses with digital clubbing.

## TECHNICAL DESCRIPTION

### Chest Radiography

**Fig. 1:**
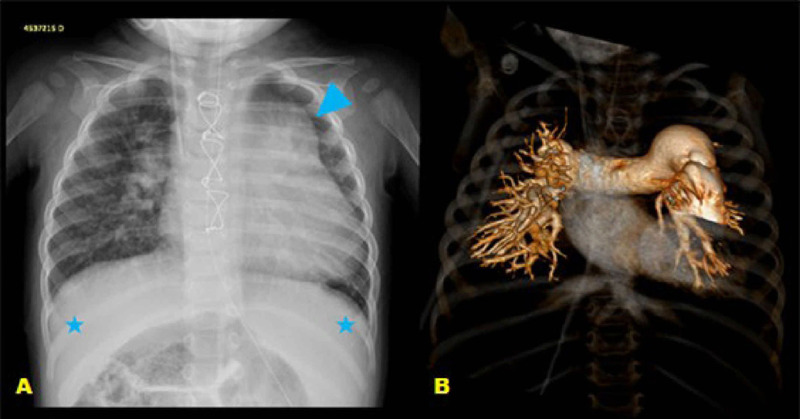
(A) Anteroposterior chest radiography showing accentuation and cephalization of the pulmonary vasculature network, and prominence of pulmonary hila with bulging of the middle arch (arrowhead) and increased cardiac area. Medialized hepatic shadow (*) occupying part of the right and left hypochondria. (B) Coronal image of the chest from a three-dimensional reconstruction of computed tomography angiography confrming that the prominence of the pulmonary hila and the bulging of the middle arch described in A correspond to the noticeably dilated pulmonary trunk and arteries.

Prominence of pulmonary vascular network and increased cardiac area with a cardiothoracic index of 0.65, suggesting congenital heart disease with increased pulmonary blood flow (Figure 1A).

### Electrocardiogram

**Fig. 2:**
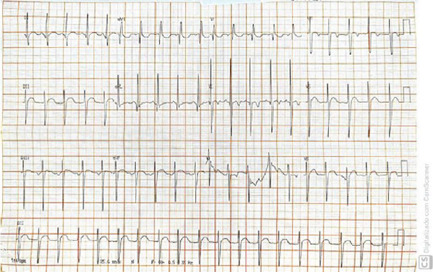
Electrocardiogram showing ectopic atrial rhythm with right ventricular overload and divisional block of the left anterosuperior branch.

Ectopic atrial rhythm, heart rate of 115 bpm, PR interval of 140 ms, SAQRS – 60°, QRS of 90 ms, suggesting right ventricular overload and left anterosuperior divisional block (Figure 2).

### Echocardiogram

*Situs solitus* in levocardia, usual venoatrial and ventriculoarterial connections, and abnormal atrioventricular (AV) connection. Single atrium with absent interatrial septum. AV connection with a single valve and two orifices, characterizing a partial AV septal defect. Moderate insufficiency of the left AV valve. Mean pulmonary artery pressure of 31 mmHg and 2.57 Qp/Qs.

Straightened interventricular septum during systole, suggesting similar pulmonary and systemic pressures. Significant dilatation of the pulmonary trunk (+9.1z) and its branches (right pulmonary artery +7.1z and left pulmonary artery +5.1z). Good biventricular function.

### Computerized Angiotomography and Venotomography

**Fig. 3:**
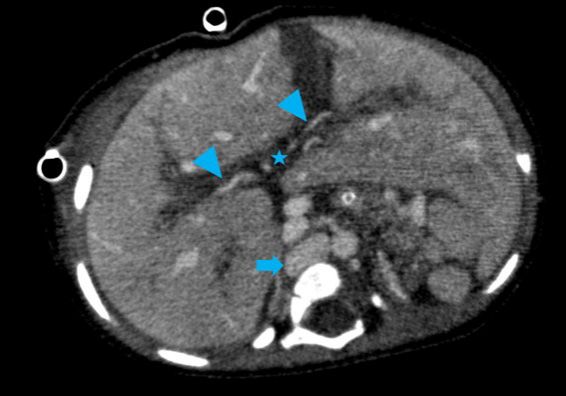
Axial image of the upper hemiabdomen in the venous phase of computed tomography showing medialized liver, inferior vena cava (solid arrow) in retrocrural position, cranially bound to the thoracic cavity with the azygos system. In the liver, the typical image of the portal vein or its branches is not identifed at the hilum level (*), identifying only the hepatic artery and ectatic intrahepatic branches (arrowheads).

**Fig. 4:**
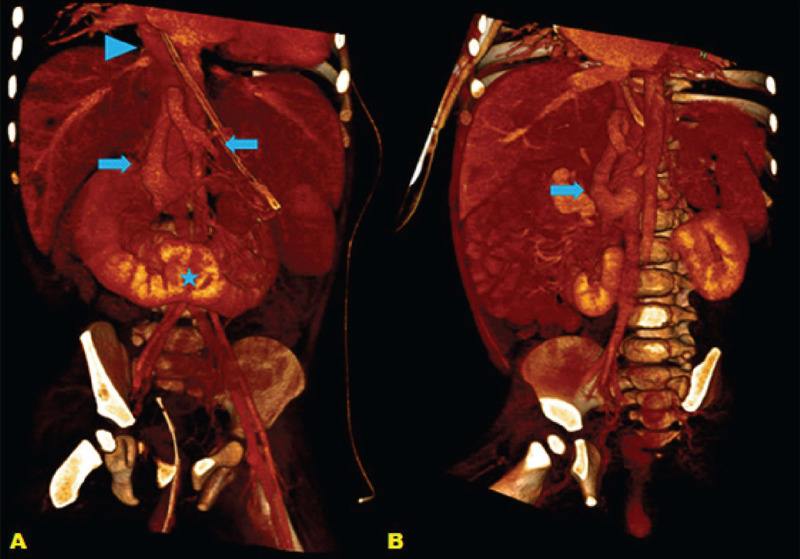
Coronal (A) and oblique (B) three-dimensional reconstructions of abdominal computed tomography, showing mesenteric-caval shunt (solid arrows) defning infrahepatic inferior vena cava engorgement, which continues with the azygos system at the thoracic level (arrowhead), best seen in A. Also, horseshoe kidneys (*) fused in the midline anterior to the abdominal aorta.

**Fig. 5:**
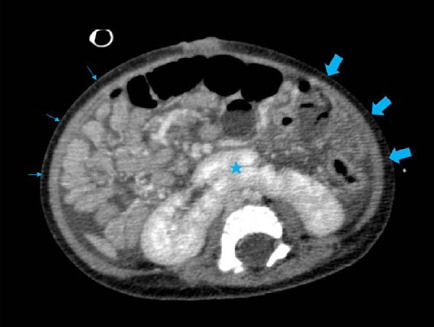
Axial computed tomography image showing horseshoe kidneys, with inferior poles (*) fused in the midline anterior to the abdominal aorta. In addition, all segments of small intestine (smoother walls and smaller caliber) positioned in their entirety in the right hemiabdomen (thin arrows), while the colonic segments are identifed only in the left hemiabdomen (thick arrows), configuring intestinal malrotation.

In the postoperative period, due to the persistence of cyanosis, further investigation and imaging study were indicated, which:

- Showed difuse dilatation of right heart chambers on qualitative analysis of pulmonary trunk and arteries (Figure 1B), aortic sinus, and ascending thoracic aorta in relation to body surface area. Apparent dilatation of right pulmonary veins and its ostia.
- Showed medialized liver and absence of inferior vena cava (IVC) intrahepatic segment. Infrahepatic portion continuing with the azygos system at the thoracic cavity level (Figure 3). Also, ectasia of the splenomesenteric venous circulation, and hepatic and superior mesenteric arteries, polysplenia, and intestinal malrotation (Figures 4 and 5).
- Not identifed the portal vein inferring agenesis, with mesenteric-caval communication at renal venous drainage level, as shown in Figure 3. There were signs suggestive of hepatic peribiliary fibrosis (at the expense of focal intrahepatic bile duct ectasia) and moderate ascites, typical findings related to Abernethy type I malformation.

## COMMENT

### Diagnosis

Due to persistent cyanosis in the immediate postoperative period, the patient underwent abdominal ultrasonography, which did not show the portal vein.

The Abernethy malformation diagnosed upon tomography represents an extrahepatic portosystemic shunt that develops between the mesenteric-portal vasculature and the systemic veins^[^1^]^. It is a rare condition that presents in association with other malformations, the most common being cardiac malformation. Anatomically, the portosystemic shunt is classifed as two types. Type 1 is characterized by complete absence of intrahepatic portal vein and a terminolateral portocaval anastomosis, a complete shunt. In type 2, the portal vein branches at the intrahepatic level are hypoplastic and patent, diverting blood from the IVC through a side-to-side shunt, therefore, a partial shunt^[^2^,^3^]^.

The portosystemic shunt alters the enterohepatic metabolism, leading to several manifestations: hyperbilirubinemia and galactosemia due to diversion of liver metabolites; hepatic encephalopathy occurs in prolonged cases; liver nodules may occur and, due to the risk of malignant degeneration, need close follow-up; ultimately, pulmonary congestion results from the resulting hepatopulmonary syndrome, leading to pulmonary hypertension and, consequently, persistent hypoxemia^[^4^,^5^]^. Hepatopulmonary syndrome is composed of the triad of liver disease, arterial hypoxemia, and pulmonary vascular dilatation. This dilation presents up to pre-capillary and capillary levels in the presence of chronic liver disease. As a result, a functional right-to-left intrapulmonary shunt occurs, with consequent arterial hypoxemia.

Three hypotheses were proposed: (i) elevation of endothelin-1 (ET-1), which increases the production of nitric oxide (NO) in the lungs, continuously stimulating NO synthase; (ii) hepatic products necessary for pulmonary vasomotor control are decreased due to hepatic dysfunction or reduced hepatic venous flow; (iii) intestinal bacteria translocation activates alveolar macrophages resulting an increase in inducible NO synthase. Therefore, elevated endotoxins due to bacterial translocation and high concentration of ET-1 in shunt blood play a contributing role in the development of hepatopulmonary syndrome^[^5^,^6^]^.

Early diagnosis was essential for follow-up and treatment, since shunt closure is possible in type 2; while in type 1, the only treatment is liver transplantation.

Thus, our confirmed type I patient, after cardiac treatment, was referred to clinical hepatology team for follow-up and possible liver transplantation.

### Operation

Surgery was performed via median sternotomy with partial resection of the right thymus. Heparinization (4 mg/kg) and bicaval and aortic cannulation. Hypothermia at 25°C with 68 minutes of cardiopulmonary bypass and nine minutes of total circulatory arrest.

Double ligation of the ductus arteriosus with 4-0 PROLENE™ threads. Right atrium opening and a single AV valve was found, with a cleft in the left AV valve without interventricular communication and absence of interatrial septum. A bovine pericardium patch was used for reconstruction of the interatrial septum, which was sutured with continuous 5-0 PROLENE™ suture.

It was chosen to keep the coronary sinus draining in the left atrium to avoid electrical conduction failure. Fenestration was maintained in the bovine pericardium patch due to history and clinical signs of pulmonary hypertension.

Cardiopulmonary bypass was finalized, and sternum was closed per protocol.

There were no surgical complications; the patient remained seven days in the pediatric cardiology intensive care unit and was discharged from the hospital on the 10^th^ day in use of diuretics and platelet anti-aggregation.
